# A novel model for Ki67 assessment in breast cancer

**DOI:** 10.1186/1746-1596-9-118

**Published:** 2014-06-16

**Authors:** Quinci Romero, Pär-Ola Bendahl, Mårten Fernö, Dorthe Grabau, Signe Borgquist

**Affiliations:** 1 Division of Oncology, Department of Clinical Sciences Lund, Lund University, SE-221 85 Lund, Sweden; 2 Department of Oncology, Skåne University Hospital, Lund, Sweden; 3 Division of Pathology, Department of Clinical Sciences, Lund University, Lund, Sweden

**Keywords:** Ki67, Breast cancer, Proliferation, Counting strategy, Statistical model

## Abstract

**Abstract:**

**Background**

Ki67 is currently the proliferation biomarker of choice, with both prognostic and predictive value in breast cancer. A lack of consensus regarding Ki67 use in pre-analytical, analytical and post-analytical practice may hinder its formal acceptance in the clinical setting.

**Methods**

One hundred breast cancer samples were stained for Ki67. A standard estimation of Ki67 using fixed denominators of 200, 400 and 1 000 counted tumor cells was performed, and a cut-off at 20% was applied, Ki67_static_. A novel stepwise counting strategy for Ki67 estimation, Ki67_scs_, was developed based on rejection regions derived from exact two-sided binomial confidence intervals for proportions. Ki67_scs_ was defined by the following parameters: the cut-off (20%), minimum (50) and maximum (400) number of tumor cells to count, increment (10) and overall significance level of the test procedure (0.05). Results from Ki67_scs_ were compared to results from the Ki67_static_ estimation with fixed denominators.

**Results**

For Ki67_scs,_ the median number of tumor cells needed to determine Ki67 status was 100; the average, 175. Among 38 highly proliferative samples_,_ the average Ki67_scs_ fraction was 45%. For these samples, the fraction decreased from 39% to 37% to 35% with static counting of 200, 400 and 1 000 cells, respectively. The largest absolute difference between the estimation methods was 23% (42% (Ki67_scs_) *vs*. 19% (Ki67_static_)) and resulted in an altered sample classification. Among the 82 unequivocal samples, 74 samples received the same classification using both Ki67_scs_ and Ki67_static_. Of the eight disparate samples, seven were classified highly proliferative by Ki67_static_ when 200 cells were counted; whereas all eight cases were classified as low proliferative when 1 000 cells were counted.

**Conclusions**

Ki67 estimation using fixed denominators may be inadequate, particularly for tumors demonstrating extensive heterogeneity. We propose a time saving stepwise counting strategy, which acknowledges small highly proliferative hot spots.

**Virtual Slides:**

The virtual slide(s) for this article can be found here: http://www.diagnosticpathology.diagnomx.eu/vs/3588156111195336

## Background

Identification of appropriate patients for adjuvant breast cancer therapies is a current challenge for medical oncologists. Optimal clinical decision making is based on both prognostic and predictive tumor markers [1]. Tumor proliferation is a cornerstone of cancer progression and is therefore a tantalizing tumor marker [2-4]. Although the mitotic index is the most established form of proliferation assessment, it has limitations because the number of mitoses per area unit is not linearly related to the rate of proliferation [5]. Cell-cycle-associated biomarkers, such as cyclin D1, cyclin E, and p21, have been considered as prognostic factors [6]. However, the net result of cell cycling is cell proliferation, and therefore immunohistochemical (IHC) analysis of Ki67 using the MIB-1 antibody has emerged as the marker of choice with both prognostic and treatment predictive value in breast cancer [7,8].

Ki67 is a nuclear non-histone protein first identified by Gerdes *et al*. in the early 1980’s at the University of Kiel, Germany. Ki67 was found to be universally expressed among proliferating cells and absent in quiescent cells, making it ripe for evaluation as a tumor proliferation biomarker [9-11]. The precise function of Ki67 remains elusive, although it is thought to be involved in ribosomal RNA synthesis [12,13]. An antibody with applicability in paraffin-embedded tissue was eventually developed and named MIB-1 for the Ki67 gene MKI67 [14].

Ki67 has shown both prognostic and predictive value in breast cancer [7,8]; however, there is an unfortunate lack of consensus regarding its use, which hinders its full clinical acceptance [15]. Significant steps have already been taken to address this issue [16]. Here, we suggest a novel strategy to optimize tumor cell evaluation that will hopefully contribute to the ongoing effort to reach an international consensus on Ki67-based assessment of proliferation.

## Methods

### Study design, patient and tumor characteristics

A retrospective cohort of fifty consecutive breast cancer patients from 2008 and 2009 with both core biopsy and corresponding surgical samples available were retrieved from the Department of Pathology, Skåne University Hospital, Lund, Sweden. The patients received no intervening anti-cancer treatment between the core biopsy and surgical excision. In total 2x50 = 100 tumor samples were included in this study. The Ethical Committee at Lund University approved the study (Dnr 529). Patient and sample characteristics have been described previously [17].

### Histopathological analyses

Representative parts of the invasive carcinoma were excised from surgical specimens and inserted into a cassette for formalin fixation. The cold ischemic time prior to excision was no longer than one hour. The needle cores were formalin-fixed immediately after extraction; the fixation times ranged from 24 to 72 hours. All specimens were paraffin-embedded following fixation. The sections were cut at 4 μm, deparaffinized, and rehydrated in graded ethanol. The antigen retrieval was performed in a microwave oven in citrate buffer pH 6 for 20 min. The expression of Ki67 was determined using the LSAB+, Dako REAL™ Detection Systems (K5001, Dako, Glostrup, Denmark). The Ki67 antibody (clone MIB-1, Dako, Glostrup, Denmark) was diluted 1:500 and incubated for 25 min in a TechMate 500 Plus (Dako, Glostrup, Denmark) and visualized with 3,3′-Diaminobenzidine. This assay method conforms to the recommendations of the International Ki67 Breast Cancer Working Group [16].

### Ki67 evaluation

First, haematoxylin and eosin (HE) stains were examined at x2 and x10 magnification to identify cancerous regions within a tissue sample. Second, the MIB-1 stain for Ki67 was examined at x2 and x10 magnification to identify hot spots, i.e., areas with an increased number of Ki67-positive cells within the previously identified cancerous regions. Finally, using x40 magnification over the hot spot, 10 cancer cells at a time were evaluated. Nuclei more brown than blue were scored positive. The number of Ki67-positive tumor cells from each set of 10 was recorded. The field of magnification was divided visually into eight “pie slices” that were evaluated from the center of the field towards the outer edge. When the entire field of magnification did not include enough cancer cells, a new field was chosen, often within the same hot spot and adjacent to the original field. If no initial hot spot could be discerned, a new field was chosen at random. Each core biopsy and surgical sample was evaluated by a single observer (QR) with the observer blinded to the relationships between the samples. Ki67 assessment was performed twice with a month in between assessments and the observer blinded to previous results.

### Model development and statistical analysis

A novel stepwise counting strategy (Ki67_scs_) was developed to assess the Ki67 status as high, low or equivocal. To evaluate Ki67_scs,_ the present study reutilized samples derived for pair-wise comparison of Ki67 levels from stained sections of pre-operative core biopsies and surgical samples [17]. Hence, the sample size of 100 was not determined by means of a power calculation. The strategy performance was evaluated using the set of all 100 samples and the sets of fifty core biopsies and fifty surgical samples separately.

**Figure 1:**
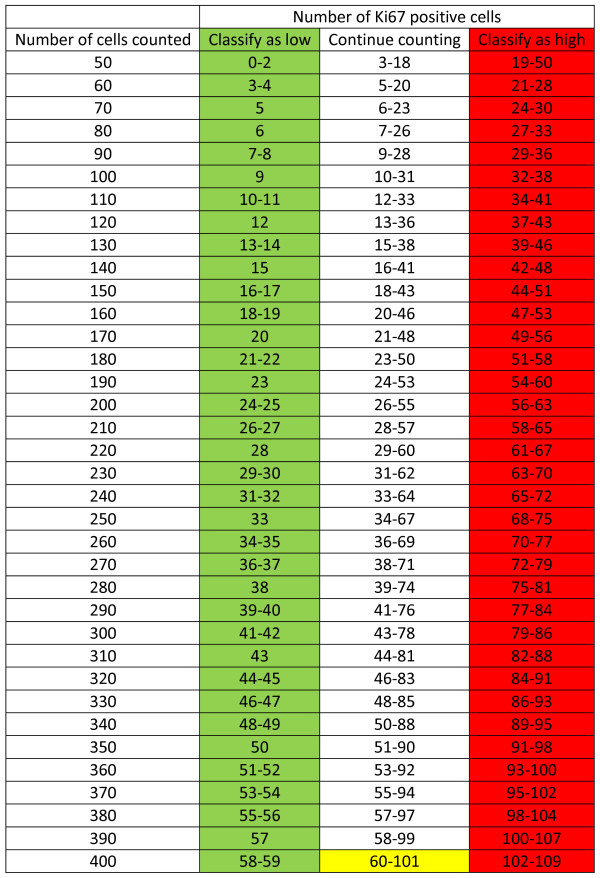
**The stepwise counting procedure in tabular form. Start by counting 50 cells in a hot spot.** If 0-2 cells are positive, declare the sample as Ki67-negative, and if 19-50 cells are positive, declare as Ki67-positive. If the number of positive cells is in the 3-18 range, count another 10 cells. If the null hypothesis was not rejected in the first step, the number of positive cells out of 60 will vary between 3+0 = 3 and 18+10 = 28. The three possible decisions based on 60, 70, …, 400 cells are listed in the table. The color-coding is green for low Ki67, red for high Ki67 and yellow for equivocal Ki67 status.

This novel strategy, with rejection regions based on two-sided exact binomial tests of the null hypothesis that the probability of Ki67-positivity is equal to a pre-specified cut-off, *c*, included the following steps 1–4:

1. A pre-determined minimum number of tumor cells (*n*_*min*_) were evaluated.

2. The resulting estimate, i.e., the fraction of Ki67 positive cells, was compared to the rejection boundaries defined below. If the estimate belonged to the upper or lower rejection region, the Ki67 status had been determined and evaluation ceased. If not, the assessment continued with step 3.

3. An additional number of tumor cells, *k* (the increment), was evaluated. It is important to choose *k* so that the difference between a predetermined maximum number of tumor cells (*n*_*max*_) and *n*_*min*_ is divisible by *k*.

4. The new cumulative estimate was compared to the corresponding rejection boundaries. If the null hypothesis could be rejected, the Ki67-status had been determined and evaluation ceased. If not, steps 3–4 were repeated until the null hypothesis was rejected, i.e., the rejection upper or lower region was reached, or until *n*_*max*_ tumor cells had been evaluated. If a rejection region was not reached after *n*_*max*_ tumor cells, then the Ki67 status of the sample was regarded as equivocal.The stepwise counting strategy for the parameters used in this study is summarized numerically in Figure 1.

The rejection regions were based on two-sided exact binomial tests of the null hypothesis that the probability of Ki67-positivity is equal to a pre-specified cut-off, *c*. The significance level, *α*_*0*_, for each test was chosen to keep the overall significance level of the test procedure at *α*. Simulation under the null hypothesis can be used to determine *α*_*0*_, a value that varies depending of the other parameters in the model, i.e. *α, c, n*_*min*_*, k,* and *n*_*max*_. The set of model parameters used in this study were: *α* = 0.05, *c* = 0.20, *n*_*min*_ = 50, *k* = 10, and *n*_*max*_ = 400.

**Figure 2:**
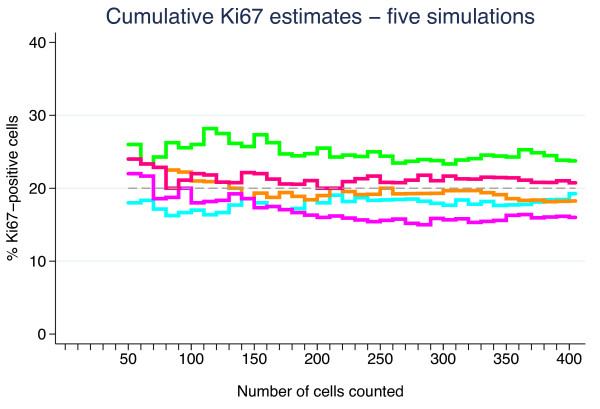
Five sequences of cumulative Ki67 fractions simulated under the null hypothesis of homogeneity and probability 0.20 of a positive cell.

**Figure 3:**
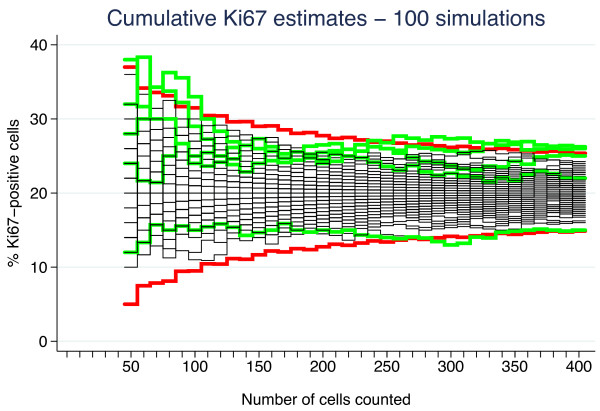
**One hundred sequences of cumulative Ki67 fractions simulated under the null hypothesis of homogeneity and probability 0.20 of a positive cell.** The red curves correspond to the upper and lower rejection boundaries based on 99.0% exact two-sided binomial confidence intervals. The five sequences that cross a boundary are highlighted in green, whereas the remaining 95 are shown in black.

To determine the appropriate significance level *α*_*0*_ of each test, a large number of random sequences of *n*_*max*_ ‘zeros’ and ‘ones’ were simulated; in these sequences, each element is ‘zero’ with probability 1-*c* and ‘one’ with probability *c*. Hence, each of the sequences corresponds to the evaluation (positive or negative) of *n*_*max*_ cells on a slide with homogeneous Ki67 staining and with probability *c* of positivity for each cell. Following the strategy described above, the simulated sequences were aggregated to cumulative fractions based on the first *n*_*min*_, *n*_*min*_ + *k*, … , *n*_*max*_-*k*, *n*_*max*_ cells. Figure 2 shows cumulative estimates from five such simulations under the null hypothesis for the set of parameter values above. Figure 3 shows 100 simulations including lower and upper rejection boundaries (red) derived from the binomial distribution. The boundaries correspond to 99.0% two-sided confidence intervals (CI). To achieve an *α* of exactly 5% for the test procedure is impossible due to the discrete nature of the test, but extensive simulation (1 000 000 sequences) has shown that by choosing *α*_*0*_ = 0.010, approximately 5% of the simulations will cross at least one of the boundaries before or at *n*_*max*_ = 400. This means that on average five of 100 simulations will falsely lead to the rejection of the null hypothesis. For the specific set of 100 randomly chosen simulations shown in Figure 3, five sequences falsely implied a conclusive Ki67 status; these sequences are highlighted in green. In total, 48 442 of the 1 000 000 simulations led to rejection of the null hypothesis, indicating that the test for this set of parameter values is slightly conservative. The statistics package Stata version 12.1 (StataCorp LP 2012, College Station, TX, USA) was used for the statistical analyses.

## Results

### Demonstration of the novel Ki67 stepwise counting strategy, Ki67_scs_

**Figure 4:**
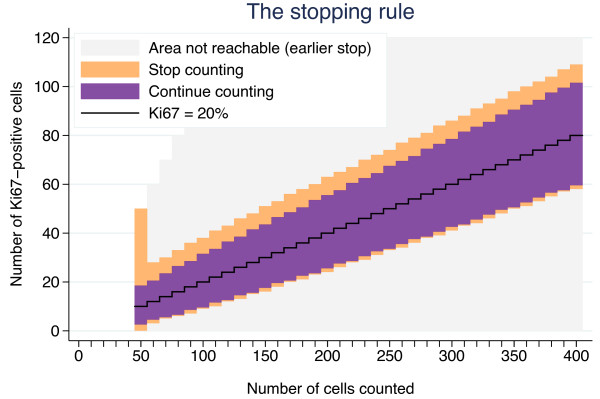
**A graphical presentation of the step-wise procedure for determination of Ki67 status.** The black jagged line corresponds to the null hypothesis of probability 0.20 of a positive cell, the cut-off. The dark region covers the counts for which the null hypothesis cannot be rejected by a two-sided binomial test at the 1.0% significance level. If the estimate falls in this region, another 10 cells are counted and a new test is performed. If 400 cells have been counted without reaching the upper or lower rejection regions (light regions), the Ki67 status of the sample is considered equivocal. If the upper or lower rejection region is reached for a sample, the counting is stopped, and the Ki67-status determined.

**Figure 5:**
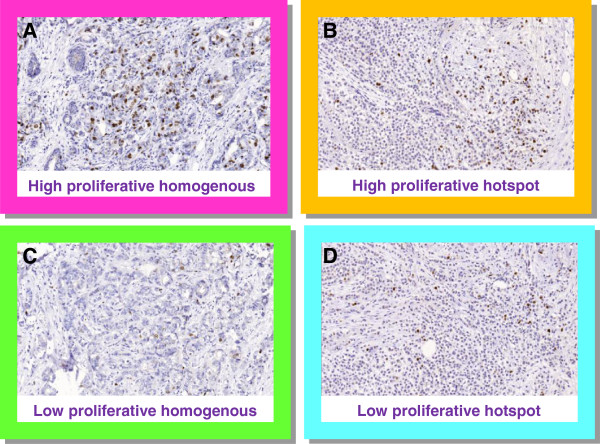
**Digital pictures at x10 magnification of four breast cancer samples stained for Ki67. A**: A highly proliferative and relatively homogenous case. **B**: A highly proliferative and heterogeneous case. **C**: A low proliferative and relatively homogenous case. **D**: A low proliferative and heterogeneous case.

The stepwise counting strategy used in this study is summarized graphically in Figure 4. Four samples depicting distinct Ki67 situations, i.e., heterogeneous and homogenous distributions for both high and low proliferative samples, were chosen to illustrate Ki67_scs_ (Figure 5). The samples’ cumulative Ki67 estimates, from 50 to 400 cells in 10 cell intervals, are shown graphically together with the boundaries of the Ki67_scs_ procedure in Figure 5. As long as a sample’s cumulative number of Ki67 positive cells was inside the band of point-wise 99.0% confidence intervals (the dark field), then another 10 cells were evaluated. If, however, the cumulative number fell outside the confidence intervals (the light field), then the Ki67 status of the sample was decided and no further tumor cells were evaluated. All four Ki67 estimates fell outside the confidence intervals, i.e., were classifiable as high (≥20%) or low (<20%) proliferative, before 200 cancer cells were evaluated. Although stopping when the rejection region is reached for the first time is recommended, cumulative estimates are shown all the way up to *n*_*max*_ = 400 tumor cells in Figure 5. Samples A, C and D remained outside the confidence intervals and maintained their proliferation status as high or low even when 400 cancer cells were evaluated. Sample B, however, changed classification from highly proliferative at 50 cells to equivocal at 400 cells. Sample B represents highly proliferative heterogeneous samples, in other words, samples with isolated hot spots.

### Comparison of Ki67_scs_ with static Ki67 counts of 200, 400 and 1 000 tumor cell sets

The Ki67 stepwise counting strategy, Ki67_scs_, was compared with static counting (Ki67_static_) of 200, 400 and 1 000 tumor cells. Using Ki67_static_, whether for 200, 400 or 1 000 tumor cells, all 100 samples were classified irrespective of the proximity of the proliferation value to the cut-off. The number of samples classified as highly proliferative decreased from 50 via 44 to 34 for 200, 400 and 1 000 cells, respectively. Of the 100 samples, 83 maintained their Ki67 status in all three static counting sets, with 34 samples consistently scoring as highly proliferative and 49 as low proliferative. Of the remaining 17 samples that did not maintain their Ki67 status, the number classified as highly proliferative using the Ki67_scs_ method decreased from 17 via 10 to one for 200, 400 and 1 000 cells, respectively using the Ki67_static_ method.

Ki67_scs_ required a median number of 100 and an average of 175 counted tumor cells to determine Ki67 status as high, low or equivocal. Thirty-two of the 100 samples were classified as high or low after the minimum number of 50 tumor cells was evaluated, three, as low and 29, as highly proliferative. Eighteen of the 100 samples were classified as equivocal when the rejection region could not be reached after the maximum number of 400 tumor cells was evaluated. Of the 82 classifiable samples, 38 were highly proliferative and 44 were low proliferative. For 74 of these 82 classifiable samples, the Ki67 status determined using Ki67_scs_ was consistent with the status determined using static sets of 200, 400 and 1 000 tumor cells. Of the remaining eight disparately classified samples, seven were highly proliferative according to either Ki67_scs_ or Ki67_static_ of 200 tumor cells. These same eight samples were all classified as low proliferative for Ki67_static_ of 1 000 tumor cells.

**Figure 6:**
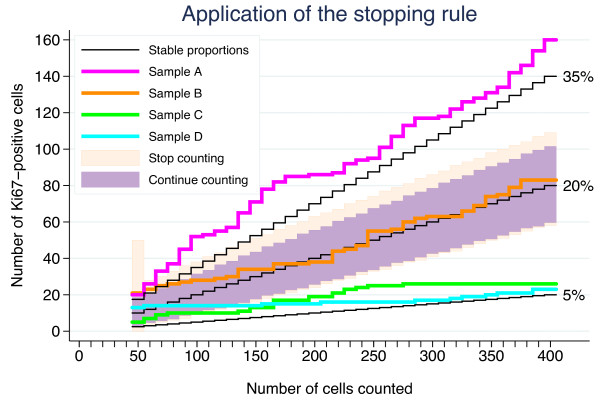
**Demonstration of the stepwise counting strategy used to determine the Ki67 status of the four cases presented in Figure**3**.**

**Figure 7:**
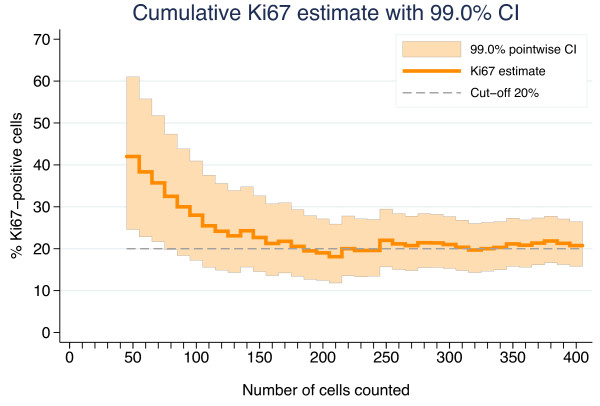
**Cumulative Ki67 estimate based on 50 to 400 tumor cells in steps of 10 for the sample (a core biopsy) showing the largest absolute and relative difference between the model-based estimate and an estimate based on the fixed counting of 200 cells.** The shaded region is a 99.0% point-wise confidence interval corresponding to the step-wise test procedure.

For the 38 samples classified as highly proliferative by Ki67_scs_, the mean Ki67-estimate (range) was 45% (26–94) compared with 39% (19–81), 37% (19–81), and 35% (16–81) using Ki67_static_ of 200, 400, and 1 000 tumor cells, respectively. The largest absolute difference between a Ki67_scs_ estimate and a Ki67_static_ estimate based on 200 cells was 23% ((42% (Ki67_scs_) *vs*. 19% (Ki67_static_)). This is more than a factor two dilution resulting in an altered Ki67 status using a cut-off at 20%. The cumulative Ki67 percentage for this sample (sample B in Figure 5 and 6) is shown in Figure 7, demonstrating the 99% point-wise CI:s from the Ki67_scs_. The Ki67 estimate at 50 cells has a CI excluding the cut-off of 20%, allowing sample classification as highly proliferative. However, when additional cells outside the initial hot spot were included in the Ki67 estimate, the value approached the cut-off, which could no longer be excluded. The sample status then changed from highly proliferative to equivocal. Further, Ki67 was evaluated separately for core biopsies and surgical samples, respectively, showing essentially the same results irrespective of the sample type (data not shown).

### Intraobserver variability

The 100 samples were evaluated twice by the same observer to assess intraobserver variability. For each of the two assessments, Ki67_scs_ was applied to the sequences of cumulative number of positive cells based on 10, 20, …, 1000 cells. In total, 78 of the samples were concordantly classified, 40 as low, 10 as equivocal and 28 as high. All but four of the remaining 22 samples were deemed equivocal based on one of the two assessments, 10 in the first assessment and 8 in the second. For the last four samples, the algorithm stopped early, after 50 to 70 cells, the second time after having detected a small hotspot which was not detected at the first assessment for which these samples were deemed Ki67 low.

## Discussion

Ki67 is the proliferation biomarker of choice in the research setting [15]; however, a lack of consensus regarding its use in pre-analytical, analytical, and post-analytical practice may hinder its formal acceptance in clinical practice [15,16]. Tissue type, warm and cold ischemic time, fixation medium and fixation time are examples of pre-analytical variables. Antibody choice, scoring method or reporting strategy are examples of analytical and post-analytical variables [16,18,19]. This study focused on the post-analytical variables, specifically the number of tumor cells evaluated and the selection of areas within a tumor section to be used for Ki67 evaluation. The analytical issues were not addressed here as only one antibody and one staining method was used.

The International Ki67 in Breast Cancer Working Group recommends scoring a minimum of 500 invasive tumor cells over at least three representative fields including proliferation zones [16]. However, among studies using Ki67, the number of tumor cells scored varies widely, ranging from tens of cells on tissue micro array cores to as many as 3,000, with a clear tendency towards the evaluation of larger sets of tumor cells [20,21]. Statistically, evaluating large numbers of cells provides smaller standard errors and therefore more accurate Ki67 estimates. For a homogenous tumor this would be true. Tumor proliferation, however, is not normally homogenously expressed [22]. Tumor samples show both intra- and intersample heterogeneity. In our previous study, the results obtained from large cell sets with narrow CI:s could provide inaccurate Ki67 values if samples showed extensive heterogeneity in proliferation [17]. Thus, heterogeneous highly proliferative tumors may be classified as low proliferative due to a dilution effect. These results suggested the need to optimize the number of tumor cells evaluated in a sample-specific manner. If the optimization could be standardized, then the intrasample heterogeneity could be accounted for statistically, and hopefully this would contribute to the ongoing effort to reach an international consensus on Ki67 assessment. In this study, adaption of the model did not seem dependent of samples type as demonstrated in analyses stratified into samples from core biopsies versus surgical samples in line with applied theoretical sampling models [23]. The sampling models discussed by Kayser *et al.*, point towards the importance of differing between random and stratified sampling, the latter requiring information of a detected object and the spatial features related to [23].

This presentation and initial evaluation of a novel Ki67 scoring methodology performed in a step-wise dynamic manner, Ki67_scs_, is based on targeting hotspots and illustrated by setting a minimum number of 50 and maximum number of 400 cancer cells to be evaluated and defining a cut-off of 20% for classifying samples as Ki67 high or low. The general practice in Ki67 scoring is based on a non-dynamic or static methodology; a pre-defined number of tumor cells are assessed and the fraction of Ki67 positive cells is determined. Thus, the novel Ki67_scs_ was compared with the standard static counting using pre-defined numbers of counted tumor cells. Ki67_scs_ is currently being developed as an open source computer program designed to enable variation of the pre-set parameters suggested and used in this study.

Five critical components of Ki67_scs_ are described here. First, the rationale for targeting hot spots is based on the assumption that regions of increased proliferation are biologically active and presumably relevant for prognosis [7,16]. High tumor proliferation as determined by Ki67 has been repeatedly demonstrated to be a negative prognostic factor [20,21,24]. In our previous study, we showed a significant risk of diluting Ki67 estimates in heterogeneous samples by including less proliferative areas of the tumor to achieve the pre-defined number of cells to be counted [17]. Thus, in this study, Ki67 evaluation was restricted to hot spots, when available. Second, an initial minimum of 50 invasive cells for Ki67 evaluation was set, presuming that a cluster of 50 highly proliferative invasive cells is enough to encourage aggressive adjuvant treatment when taken together with supplementary clinical and tumor features. We recognize that this is a subjective judgment and propose that this lower limit be adjustable within the Ki67_scs_ program. Third, a maximum of 400 invasive cells for Ki67 evaluation was set; this number was based on a doubling of the Swedish clinical practice of evaluating 200 cells. We acknowledge that The International Ki67 in Breast Cancer Working Group working group recommends a minimum of 500 cancer cells for Ki67 evaluation. This recommendation, however, is not based on the use of hot spots as suggested above but on representative averages and is dependent on sample type [16]. In this study, we chose to designate cases requiring more than 400 tumor cells for classification as equivocal. In clinical practice, these cases would employ other factors to guide treatment choice. An exact cut-off, although attractive in theory, is not considered feasible in practice due to methodological limitations. Ideally, no fixed upper limit should exist. Just as the number of tumor cells evaluated needs to be optimized for each sample based on its individual heterogeneity, the upper limit should be flexible. Theoretically, homogeneous samples tolerate a higher upper limit, whereas highly heterogeneous samples may require a much lower upper limit to avoid dilution. Therefore, the upper limit was set as an adjustable parameter within the Ki67_scs_ program. Fourth, a cut-off of 20% was set for classification of samples as high or low proliferative based on South-Swedish clinical practice and as discussed in our previous work [17]. The literature conveys a plethora of cut-off values, although cut-offs in the 10%–20% range are most commonly used to dichotomize Ki67 values [20,25]. Deprived of standardization, cut-offs have limited value outside the studies and centers from which they originated. Furthermore, cut-offs are context-related, e.g., a value appropriate for determination of prognosis may not be relevant for determination of trial eligibility or for use as a pharmaco-dynamic marker. We suggest the cut-off value should be adjustable within the Ki67_scs_ program. Standardization of Ki67 cut-off values for different breast cancer types and study goals is an important future challenge. Fifth, the type I error *α* of the stepwise procedure was set to 5%. The stepwise procedure will meet this significance level for homogenous samples, but it is not clear what *α* will be when the assumption of homogeneity is violated, i.e. for heterogeneous samples. It will most likely be larger, but the truth regarding the Ki67 status of samples with small but highly positive hotspots is unknown. This well-defined and simple stepwise method will pinpoint some samples as positive which would have been regarded as negative if a large static number of cells had been counted. Hence the parameter *α* should be seen rather as a tuning parameter than a true type I error. The aim of Ki67scs is to enable cessation of tumor cell evaluation as soon as a reliable classification is achieved to reduce the risk of a dilution effect. As an initial demonstration of Ki67_scs_, we analyzed four cases representing heterogeneous and homogenous Ki67 distributions for both high and low proliferative samples, as illustrated in Figure 4. As shown in Figure 6, all four samples were classified based on fewer than 150 tumor cells using Ki67_scs_, and samples A, C and D maintained their Ki67_scs_ classification at 200 and 400 cells. Figure 7 shows an example of an isolated hot spot that was classified as highly proliferative after counting only 50 cancer cells. As more cells were evaluated, however, the Ki67 estimate dropped considerably, from 40% to less than 20% at 200 cells counted. This illustrates how a dilution effect can alter a classification from high to low. The challenges regarding a fixed cut-off should be noted. An exact cut-off, although attractive in theory, may not be feasible in practice due to methodological limitations. When a sample’s Ki67 is too close to the chosen cut-off it should be categorized as equivocal and other clinic-pathological variables should be taken into account. This study is the first to report on a novel method for Ki67 assessment and we recognize that prior to application in the clinic, additional improvements are needed, i.e. studies in a larger cohort assessing the prognostic/predictive value of the equivocal grouping evaluated in order to reach for a “gold standard”.

To further test Ki67_scs_, we compared the results from the 100 breast cancer samples, 50 core biopsies and 50 surgical samples with static counting of 200, 400 and 1 000 cells. The number of highly proliferative samples decreased across the 200, 400 and 1 000 sets, suggesting a dilution. Using Ki67_scs_, the samples were classified according to a 20% cut-off as Ki67 high, low or equivocal. Interestingly, the average Ki67 value for the highly proliferative samples was ten percentage units lower using Ki67_static_ with 1 000 cells than Ki67_scs_ (35% *vs*. 45%). Larger individual variations were noted, with an absolute maximum decrease of 23% for a single sample.

Automated counting procedures have been investigated in previous publications addressing the utility for Ki67 assessment [26,27]. In the work by Fasanella *et al.*, the authors describe discrepancy in Ki67 results between automated assessment and human evaluation revealing higher Ki67 values in the latter [27]. Mohammed *et al.*, however report excellent agreement between automated and visual Ki67 labeling index. As a prognostic tool both methods were useful, however the visual method being superior [26]. This study has not addressed automated Ki67 assessment; however the proposed counting model should have no limitations favoring either human/visual or automated counting.

The definition of truth as for Ki67 levels is theoretically interesting, and sums up the ongoing international discussion on Ki67 assessment. The “true” Ki67 level may theoretically be the level derived from a certain assessment method that would depict the most appropriate prognostic or treatment predictive value. This paper, however, was not designed to solve this question, and future studies with long-term follow-up comparing the static and the sequential method, may be able to narrow down the most optimal assessment method.

## Conclusions

To summarize, for Ki67 assessment in breast cancer, static counting of tumor cells may lead to a diluted Ki67 estimate with the risk of misclassifying a sample, particularly when heterogeneous and highly proliferative samples are evaluated. The stepwise counting strategy presented herein may reduce the risk of diluting the Ki67 estimate. Attempting to optimize the number of invasive cancer cells assessed for each sample allows for sample heterogeneity and hopefully contributes to the current consensus discussion regarding Ki67 evaluation. Future studies are needed to validate our model in an independent dataset, address the prognostic value of the suggested Ki67 assessment method, and to test inter-observer agreement with this novel strategy.

## Abbreviations

CI: Confidence interval; HE: Haematoxylin and Eosin; IHC: Immunohistochemistry; Ki67_scs_: Ki67 stepwise counting strategy; Ki67_static_: Ki67 static counting strategy.

## Competing interests

The authors declare that they have no competing interests.

## Authors’ contributions

QR and DG carried out the pathological assessments. PB participated in the design of the study and performed the statistical analysis. SB, QR, and PB conceived of the study, and all authors participated in its design and coordination and helped to draft the manuscript. QR, PB and SB drafted the manuscript. All authors read and approved the final manuscript.
